# Induction of Mucosal Humoral Immunity by Subcutaneous Injection of an Oil-emulsion Vaccine against *Salmonella enterica* subsp. *enterica* serovar Enteritidis in Chickens

**DOI:** 10.14252/foodsafetyfscj.2018003

**Published:** 2018-11-20

**Authors:** Yuuichi Ishida, Eishi Sakai, Katsuo Sato, Einori Sugiyama, Kazuyuki Mima, Akira Taneno, Hirofumi Shimomura, Longzhu Cui, Yoshikazu Hirai

**Affiliations:** 1 Division of Bacteriology, Department of Infection and Immunity, Jichi Medical University, 3311-1, Yakushiji, Shimotsuke-shi, Tochigi 329-0498, Japan; 2 Choka Research Institute, vaxxinova Japan K.K., 809, Choka, Nikko-shi, Tochigi 321-1103, Japan; 3 Department of Nutritional Science, Faculty of Human Life Science, Shokei University, 2-6-78, Kuhonji, Chuo-ku, Kumamoto-shi, Kumamoto 862-8678, Japan; 4 Tamano Institute of Health and Human Services, 1-1-20 Chikko Tamano-shi, Okayama 706-0002, Japan

**Keywords:** bacterial attachment, inactivated oil-emulsion SE vaccine, mucosal humoral immunity, *Salmonella* Enteritidis

## Abstract

*Salmonella enterica* subsp. *enterica* serovar Enteritidis (SE) is one of the major causes of food poisoning. Much effort has been made to develop a vaccine for the prevention of SE colonization and infection in poultry. However, the effect of inactivated whole-cell SE vaccines on the bacterial attachment has not been clarified. This study investigated the immune responses to a killed whole-cell SE vaccine in chickens and the effect of vaccination on the bacterial attachment of SE to cultured Vero cells. A 1 ml dose of 10^8^–10^9^ CFU viable SE bacterial cells was orally administered to chickens at 4 weeks or 10 months post vaccination. The number (CFU) of SE in 1 g of cecal droppings was counted on day 6 after administration. The SE CFUs were significantly lower (*p* < 0.05) in the vaccinated chickens, not only at 4 weeks but also at 10 months after vaccination, than in the unvaccinated control chickens. Anti-SE IgG and anti-SE IgA were detected using enzyme-linked immunosorbent assay (ELISA) in serum and intestinal and oviduct fluid samples from vaccinated chickens. Adhesion of heat-killed SE cells to Vero cells was reduced by pre-treatment of the bacteria by the vaccinated chicken-derived intestinal fluid, indicating the potential of the vaccine-induced antibody to prevent SE adhesion to epithelial cell surfaces.

## 1. Introduction

*Salmonella* is a gram-negative bacterium that can live in the intestinal tract of humans and various animals, and cause food poisoning in humans accompanying symptoms such as diarrhea. It may invade the bloodstream and cause systemic symptoms such as sepsis^1^^)^. Eggs and poultry products contaminated with the *Salmonella* species, often *S. enterica* subsp. *enterica* serovar Enteritidis (SE), are a major cause of food poisoning. Worldwide, there are ongoing efforts to develop a vaccine for the prevention of SE colonization and infection in poultry.

*Salmonella* infections are initiated by bacterial attachment to and colonization at the infection site. The inhibition of adhesion is seen as a way of preventing SE infections^2^^,^^3^^)^. *In vitro* studies reported that outer membrane protein (OMP)-specific IgY inhibited the attachment of SE to Caco-2 human epithelial colorectal adenocarcinoma^2^^)^, and that the entry of SE into human epithelial type 2 (HEp-2) cells was suppressed by an SE-specific antibody^4^^)^. The adhesion of SE to ovarian granulosa cells was shown to be suppressed by an anti-chicken fibronectin antibody^3^^)^. Cultured Vero African green monkey kidney epithelial cells have also been used in studies on *Salmonella* invasion^5^^)^.

Various types of *Salmonella* vaccines have been evaluated, including one that used bacterial ghosts as the antigen^6^^)^. The tested vaccines have induced specific IgG and IgA, cellular immunity with increases in CD4^+^ and CD8^+^ T cells, and reduction of egg contamination^1^^)^. No reports of the immunogenicity of inactivated whole-cell SE vaccines are available. This study investigated the immune responses to a killed whole-cell SE vaccine in chickens and the effect of vaccination on the bacterial attachment of SE to cultured Vero cells.

## 2. Materials and Methods

### 2-1. Type of Chicken

Female white leghorn chickens hatched from specific pathogen free (SPF) eggs (Australian SPF Services, Woodend, Australia) were used. No *Salmonella* species were detected in the feces of the chickens prior to the start of the study. The experimental procedures and animal management protocols complied with the Basic Guidelines on Animal Experiments etc. in Research Institutions etc. Supervised by the Ministry of Agriculture, Forestry and Fisheries in Japan.

### 2-2. Salmonella

*S. enterica* subsp. *enterica* serovar Enteritidis (SE) strain rifHY-1 was provided by Dr. M. Nakamura at Kitasato University (Towada, Japan) and was maintained in our laboratory. This strain is a rifampicin-resistant mutant.

### 2-3. Vaccination

A commercially available oil-emulsion inactivated SE vaccine (AviPro 109 SE4; Lohmann Animal Health International, Winslow, ME, U.S.A.) was used. Vaccination was by subcutaneous injection of 0.25 ml of vaccine at the age of 5 and 22 week-old.

### 2-4. Experimental Infection of SE and Measurement of Colony-forming Units (CFUs)

Four weeks or 10 months after vaccination into 5 week-old chickens, ten chickens were given an oral dose of 1 ml bacterial suspension containing 10^8^ to 10^9^ CFU/ml SE. Ten unvaccinated chickens were controls. Cecal droppings were collected 6 days after the SE administration, 1 g wet weight samples were homogenized in 10 ml of Hanja tetrathionate (HTT) broth (Eiken Chemical, Tokyo, Japan). Tenfold serial dilutions of 25 µl aliquots in phosphate buffered saline (PBS) were inoculated on deoxycholate hydrogen sulfide lactose agar plates (Eiken Chemical, Tokyo, Japan) containing 100 µg/ml rifampicin and incubated for 24 h at 37°C under aerobic conditions. Bacterial colonies were counted and the log_10_ CFU/g was calculated. Regarding the “4 weeks after vaccination” experiment, 3 batches of the vaccine were tried. And regarding the “10 months after vaccination” experiment, 1 batch was tried.

### 2-5. Preparation of Samples

At 26 weeks of age, namely 4 weeks after vaccination into 22 week-old chickens, sera were collected, 10-cm lengths of small intestine and oviduct were aseptically excised from 5 chickens and the mucus was collected by washing with 5 ml PBS containing 0.67% bovine serum albumin. The mucosal fluid was diluted approximately ten-fold during collection. The samples were vortexed for 10 sec, centrifuged for 10 min at 5,000 *g*, and passed through a 1.0 µm pore size filter.

### 2-6. Detection of Antibodies against SE

The mucosal fluid and 400-fold diluted serum samples were pipetted into Nunc MaxiSorp flat bottom 96-well plates (Thermo Fisher Scientific, Rochester, NY, USA) coated with killed SE cells and incubated for 2 h at 37°C. Horseradish peroxidase-conjugated goat anti-chicken IgG or anti-chicken IgA (Bethyl Laboratories, Montgomery, TX, USA) were added, and after reacting with tetramethylbenzidine (TMB) enzyme-linked immunosorbent assay (ELISA) substrate solution (SureBlue Reserve, KPL/SeraCare, Milford, MA, USA) absorbance at 450 nm (A_450 nm_) was measured in triplicate.

### 2-7. Assay of Adhesion of Heat-killed SE to Vero Cells

Since there are no comparable bird epithelial cell culture lines, Vero mammalian kidney epithelial cells were used in the cell culture assays. The SE suspensions were washed with PBS and collected by centrifugation at 10,000 *g* for 10 min, resuspended in PBS, and heat inactivated at 80°C for 10 min to prevent the growth of viable bacterial cells and self-aggregation during the experimental procedures.

Vero cells were cultured in 60 x 15 mm dishes (Becton Dickinson and Company, Franklin Lakes, NJ, USA) in RPMI 1640 (Wako Pure Chemical Industries, Osaka, Japan) at 37°C in a humidified incubator with 5% CO_2_. Suspensions of heat-killed bacteria (A_660_ = 0.15) were incubated for 1 h at 37°C with 100-fold diluted serum or tenfold diluted small intestinal mucosal fluid, added to Vero cell culture monolayers and incubated for 30 min 37°C and 5% CO_2_. The Vero cells were rinsed twice with PBS to wash away nonadherent SE, and the stained with Wright–Giemza solution for 15 min at room temperature. Bacterial cells adherent to 50 randomly selected Vero cells were observed at 1,000-fold magnification and the mean number of SE bacteria per Vero cell was calculated.

### 2-8. Statistics

The significance of the differences in the numbers of SE bacteria in cecal droppings, in the SE-specific antibody levels and in the numbers of heat-killed SE adhered to Vero cells was evaluated by student’s *t*-test.

## 3. Results and Discussion

**Fig. 1.**
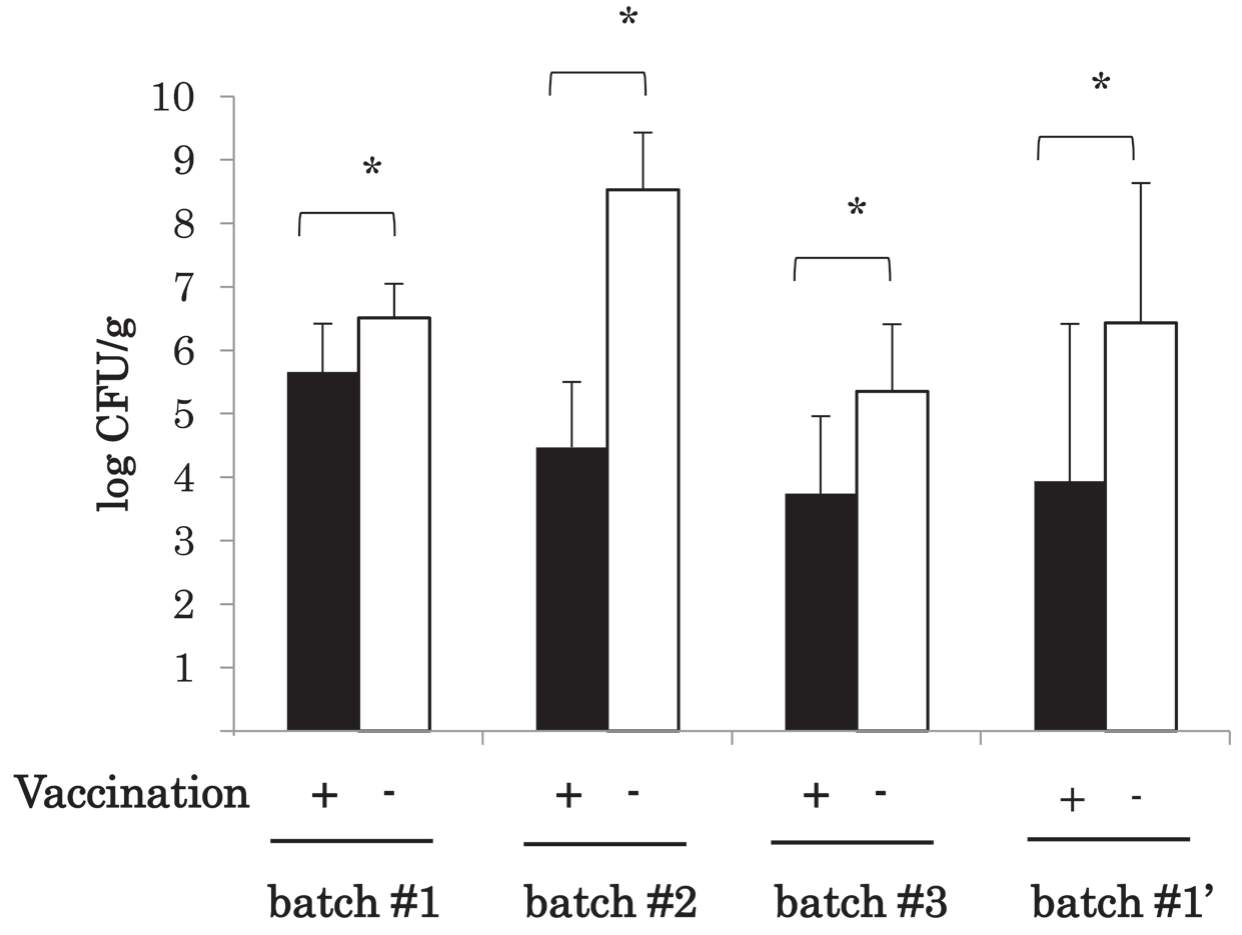
Comparison of the number (CFU) of SE in cecal droppings of chickens which were vaccinated and not vaccinated. CFUs obtained from 10 samples are represented as mean CFU ± SD. Asterisks indicate statistical significance (*p* < 0.05).

The number of SE bacteria in cecal droppings from the vaccinated chickens was significantly (*p* < 0.05) lower than in the unvaccinated chickens not only at 4 weeks (Fig. 1, batch #1 - #3) but also at 10 months after vaccination (Fig. 1, batch #1’). The effect persisted for at least 10 months. Therefore, we consider that the SE vaccine possesses the effect of clear reduction of bacteria shedding, namely, the reduction of SE colonization. Similar shedding reduction experiments using another commercially available SE inactivated vaccine, viable bacteria of O9, O4 and O7 group have been reported^7^^)^. In those experiments, the vaccine partially showed cross protection against O4 group, but did not against O7 group. We are planning to conduct similar adhesion reduction experiments using bacteria of other serotypes including these ones and to study the wide potential of this vaccine.

**Fig. 2.**
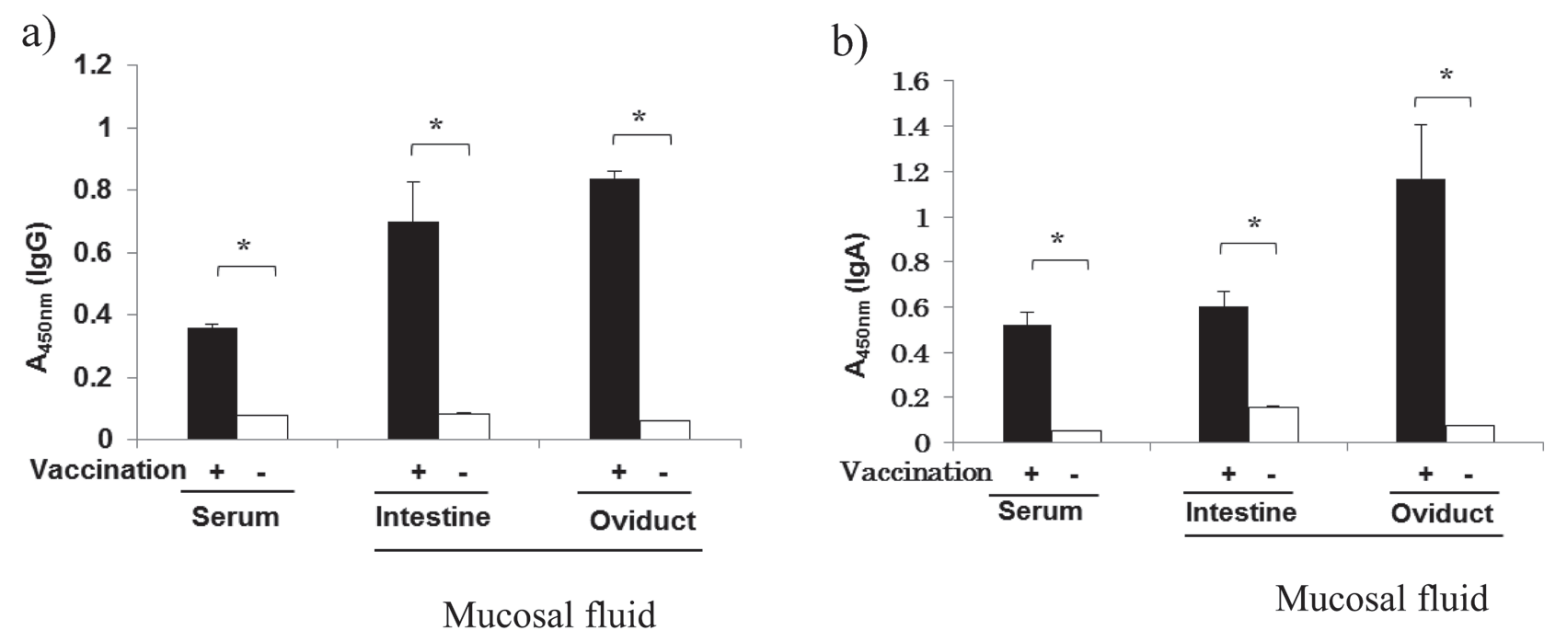
Level of antibodies specific to SE in vaccinated chickens. A450 nm values are the means ± SD of the absorbance measured in five samples. Asterisks indicate statistical significance (*p* < 0.05).

At 4 weeks after vaccination into 22 week-old chickens, anti-SE IgG and IgA levels in 400-fold diluted sera and in ten-fold dilutions of the fluid collected from the small intestine and oviduct were significantly higher in the vaccinated chickens than in the unvaccinated controls (Fig. 2 (a) and (b)). IgA is involved in mucosal immunity in mammals and birds^8^^,^^9^^)^. Pathogen-specific IgG antibody has been reported in the trachea and intestine in chickens following the stimulation of mucosal antibody producing cells by an inactivated Newcastle disease vaccine^10^^)^. In this study, anti-SE IgG was found in the intestinal mucosa washings, but the secretion mechanism is not clear, and the ways in which IgG and IgA are secreted from human intestinal mucosa may differ^11^^)^.

**Fig. 3.**
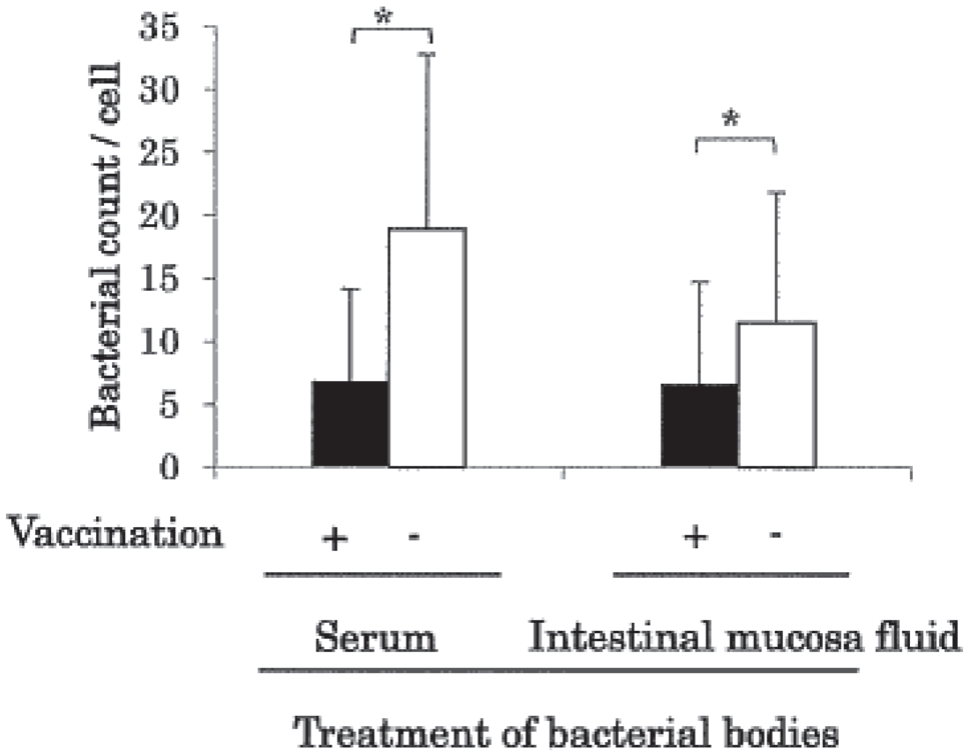
Inhibition effect of vaccinated chicken serum and intestinal mucosal fluid on the adhesion of SE to Vero cells. The data are mean SE/Vero cell ± SD from three independent experiments. Asterisks indicate statistical significance (*p* < 0.05).

Treatment of heat-killed SE with the sera and intestinal mucosal fluid from the vaccinated chickens significantly (*p* < 0.05) reduced its adhesion to Vero cells (Fig. 3). The results suggest that the reduced adhesion of SE to intestinal mucosa contributed to the decrease in bacterial colonization seen in vaccinated chickens. It was not confirmed that the anti-SE IgG and IgA mediated the reduced adhesion of SE to the Vero cells. A study of adhesion using intestinal fluid, from which IgA or IgG is absorbed, is planned.

SE contamination of eggs in the ovaries and oviducts ultimately results not only in food poisoning but also in significant economic losses in the poultry industry^12^^)^. In this study, oral inoculation of viable SE-infected unvaccinated chickens, a large number of viable SE were isolated from the intestinal tract, but not from the oviduct. As a previous study reported similar results^13^^)^, oral SE exposure may easily produce intestinal but not oviduct colonization. Viable SE has been recovered from the chicken ovaries and oviduct following intravenous inoculation^14^^)^, indicating that in the presence of bacteremia, SE may reach the ovaries and oviduct. Humoral SE immunity has been found to protect against SE contamination of eggs in the oviduct^15^^)^. Significant increases in anti-SE IgA and IgG were observed in both the intestinal and oviduct mucosa of vaccinated chickens, indicating that if viable SE cells were to reach the ovaries and oviduct, the vaccine would reduce the likelihood of colonization. In addition, in vaccinated chickens, the high anti-SE-IgG titer detected in the blood would be expected to protect against bacteremia.

In conclusion, this commercially available inactivated SE oil-emulsion vaccine reduced excretion of SE in cecal droppings, increased anti-SE-IgA and IgG in the serum and intestinal oviduct mucosa, and reduced SE adhesion to epithelial (Vero) cells. The development of novel SE vaccines is ongoing. These results, particularly those relating to adhesion of SE to epithelial cells, are expected to assist in that effort.

## References

[1] Peng W, Si W, Yin L, et al. *Salmonella enteritidis* ghost vaccine induces effective protection against lethal challenge in specific-pathogen-free chicks. Immunobiology. 2011; 216: 558–565. 10.1016/j.imbio.2010.10.001 21247655

[2] Chalghoumi R, Théwis A, Beckers Y, Marcq C, Portetelle D, Schneider YJ. Adhesion and growth inhibitory effect of chicken egg yolk antibody (IgY) on *Salmonella enterica* serovars Enteritidis and Typhimurium *in vitro*. Foodborne Pathogens and Disease. 2009; 6: 593–604. 10.1089/fpd.2008.0258 19388827

[3] Thiagarajan D, Saeed M, Turek J, Asem E. In vitro attachment and invasion of chicken ovarian granulosa cells by *Salmonella enteritidis* phage type 8. Infect Immun. 1996; 64: 5015–5021. 8945540 10.1128/iai.64.12.5015-5021.1996 PMC174482

[4] Iankov ID, Petrov DP, Mladenov IV, Haralambieva IH, Mitov IG. Lipopolysaccharide-specific but not anti-flagellar immunoglobulin A monoclonal antibodies prevent *Salmonella enterica* serotype enteritidis invasion and replication within HEp-2 cell monolayers. Infect Immun. 2002; 70: 1615–1618. 10.1128/IAI.70.3.1615-1618.2002 11854252 PMC127784

[5] Barrow PA, Lovell MA. Invasion of Vero cells by *Salmonella* species. Journal of Medical Microbiology. 1989; 28: 59–67. 10.1099/00222615-28-1-59 2643706

[6] Hajam IA, Dar PA, Won G, Lee JH. Bacterial ghosts as adjuvants: mechanisms and potential. Veterinary Research. 2017; 48: 37. 10.1186/s13567-017-0442-5 28645300 PMC5482964

[7] Nakamura M, Nishimura H, Nagata T, Takehara K The effect of *Salmonella* Enteritidis Inactivated Vaccine on Shedding of O9, O4,O7 Serovars of *Salmonella*. J Jpn Soc Poultry Dis. 2002; 38: 149–152.

[8] Blutt SE, Miller AD, Salmon SL, Metzger DW, Conner ME. IgA is important for clearance and critical for protection from rotavirus infection. Mucosal Immunology. 2012; 5: 712–719. 10.1038/mi.2012.51 22739233 PMC3461240

[9] Sun Q, Shang Y, She R, et al. Detection of intestinal intraepithelial lymphocytes, goblet cells and secretory IgA in the intestinal mucosa during Newcastle disease virus infection. Avian Pathol. 2013; 42: 541–545. 10.1080/03079457.2013.845292 24087844

[10] Chimeno Zoth S, Gómez E, Carrillo E, Berinstein A. Locally produced mucosal IgG in chickens immunized with conventional vaccines for Newcastle disease virus. Brazilian Journal of Medical and Biological Research. 2008; 41: 318–323. 10.1590/S0100-879X2008000400010 18392454

[11] Yoshida M, Claypool SM, Wagner JS, et al. Human neonatal Fc receptor mediates transport of IgG into luminal secretions for delivery of antigens to mucosal dendritic cells. Immunity. 2004; 20: 769–783. 10.1016/j.immuni.2004.05.007 15189741

[12] De Buck J, Van Immerseel F, Haesebrouck F, Ducatelle R. Colonization of the chicken reproductive tract and egg contamination by *Salmonella*. J Appl Microbiol. 2004; 97: 233–245. 10.1111/j.1365-2672.2004.02294.x 15239689

[13] Nakamura M, Nagamine N, Takahashi T, Suzuki S, Sato S. Evaluation of the efficacy of a bacterin against *Salmonella* enteritidis infection and the effect of stress after vaccination. Avian Diseases. 1994; 38: 717–724. 10.2307/1592106 7702503

[14] Woodward MJ, Gettinby G, Breslin MF, Corkish JD, Houghton S. The efficacy of Salenvac, a *Salmonella enterica subsp*. Enterica serotype Enteritidis iron-restricted bacterin vaccine, in laying chickens. Avian Pathol. 2002; 31: 383–392. 10.1080/03079450220141660 12396340

[15] Withanage GSK, Sasai K, Fukata T, Miyamoto T, Baba E. Secretion of *Salmonella*-specific antibodies in the oviducts of hens experimentally infected with *Salmonella* enteritidis. Vet Immunol Immunopathol. 1999; 67: 185–193. 10.1016/S0165-2427(98)00223-2 10077424

