# Author Correction: Induction of Mucosal Humoral Immunity by Subcutaneous Injection of an Oil-emulsion Vaccine against *Salmonella Enterica* subsp. *enterica* serovar Enteritidis in Chickens

**DOI:** 10.14252/foodsafetyfscj.2018020

**Published:** 2019-06-28

**Authors:** Yuuichi Ishida, Eishi Sakai, Katsuo Sato, Einori Sugiyama, Kazuyuki Mima, Akira Taneno, Hirofumi Shimomura, Longzhu Cui, Yoshikazu Hirai

**Affiliations:** 1 Division of Bacteriology, Department of Infection and Immunity, Jichi Medical University, 3311-1, Yakushiji, Shimotsuke-shi, Tochigi 329-0498, Japan; 2 Choka Research Institute, vaxxinova Japan K.K., 809, Choka, Nikko-shi, Tochigi 321-1103, Japan; 3 Department of Nutritional Science, Faculty of Human Life Science, Shokei University, 2-6-78, Kuhonji, Chuo-ku, Kumamoto-shi, Kumamoto, 862-8678, Japan; 4 Tamano Institute of Health and Human Services, 1-1-20 Chikko Tamano-shi, Okayama 706-0002, Japan

## Abstract

To the Editor, in November 2018, we published a *Short Communication* on humoral immunity induction against *Salmonella* in chickens. We are writing to inform errors on statistics. Our principal conclusions, however, remain unchanged.

Original publication “Induction of mucosal humoral immunity by subcutaneous injection of an oil-emulsion vaccine against *Salmonella enterica* subsp. *enterica* serovar Enteritidis in chickens” in 2018. Vol. 6, No. 4, 151–155, https://doi.org/10.14252/foodsafetyfscj.2018003, published online 20 November 2018.

The authors regret any confusion or inconvenience caused by these errors.

**Page 152:**

In the “2-3. Vaccination” section, the vaccine name “AviPro 109 SE4” was incorrect. The name should read “AviPro 109 SE4C”.

**Page 153:**

The results shown in Fig. 1. has been re-evaluated by use of a more adequate statistics method, Wilcoxon-Mann-Whitney test.

**Fig. 1:**
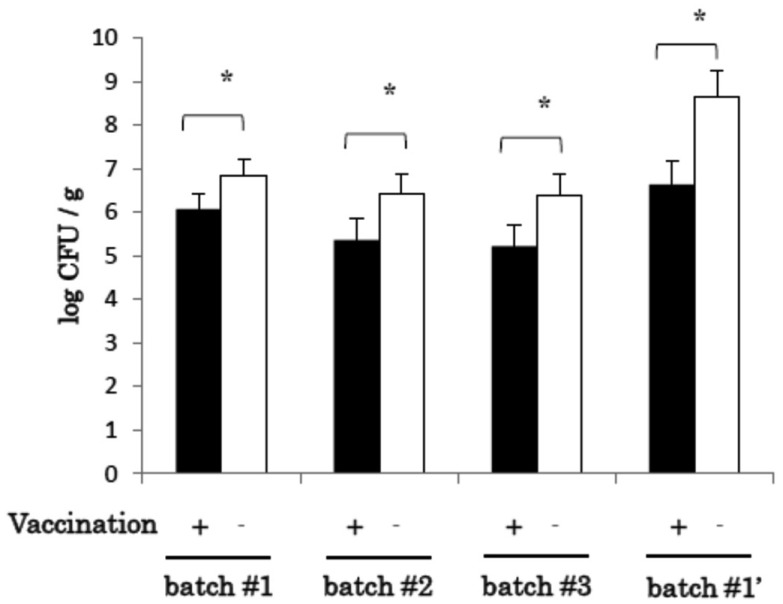


Regarding this change, the whole description of the “2-8. Statistics” section should be ;

“The significance of the differences in the numbers of SE bacteria in cecal droppings was evaluated by Wilcoxon-Mann-Whitney test. The significance of the differences in the SE-specific antibody levels and in the numbers of heat-killed SE adhered to Vero cells was evaluated by Student’s *t*-test.”

**Page 154:**

In the legend of **Fig. 2.**, the description of “means ± SD” was incorrect. The correct one is “means ± SE”, i.e., standard error.

In Fig. 3., “Intestinal mucosa fluid” in the x-axis was incorrect. It should read “Intestinal mucosal fluid”. Besides, “Treatment of bacterial bodies” should be shortened to “Treatment” and moved to just under “Vaccination”.

**Fig. 3:**
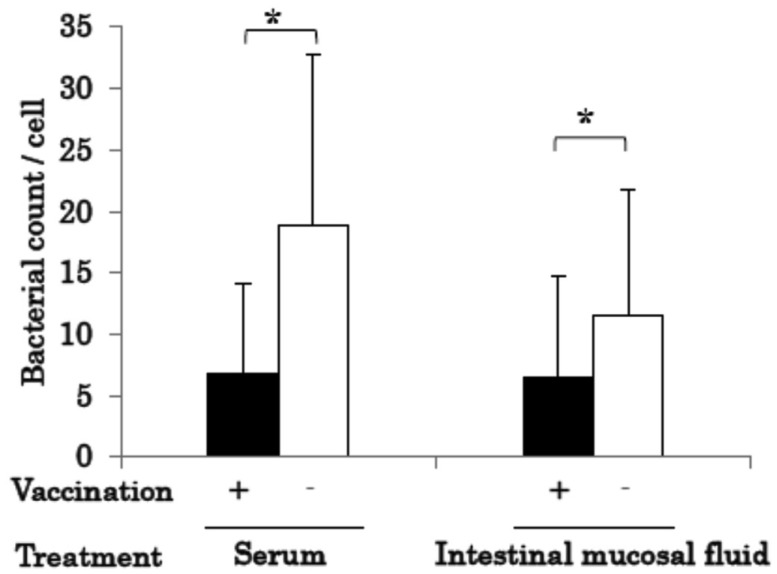


These changes do not affect the conclusions of this work.

